# A 10‐Year‐Old Boy With Chronic Atrial and Intestinal Dysrhythmia (CAID) and Chronic Intestinal Pseudo‐Obstruction (CIPO)

**DOI:** 10.1002/ccr3.72111

**Published:** 2026-02-28

**Authors:** Amirhossein Hosseini, Mina Alibeik, Soheil Omid, Koroush Vahidshahi, Soheila Vaghefi, Aliakbar Sayyari, Farid Imanzadeh

**Affiliations:** ^1^ Pediatric Gastroenterology, Pediatric Gastroenterology, Hepatology and Nutrition Research Center, Research Institute for Children's Health Shahid Beheshti University of Medical Sciences Tehran Iran; ^2^ Research Institute for Children's Health Shahid Beheshti University of Medical Sciences Tehran Iran; ^3^ Shahid Modarres Hospital, Faculty of Medicine Shahid Beheshti University of Medical Sciences Tehran Iran; ^4^ Mofid Children's Hospital, Department of Forensic Medicine and Toxicology, School of Medicine Shahid Beheshti University of Medical Sciences Tehran Iran

**Keywords:** chronic atrial and intestinal dysrhythmia (CAID), chronic intestinal pseudo‐obstruction (CIPO), pediatric, SGO

## Abstract

Chronic intestinal pseudo‐obstruction (CIPO) is a severe gastrointestinal syndrome characterized by disruption of normal gut movement, resembling a mechanical obstruction, despite the absence of any physical obstruction. It can occur idiopathically or as a secondary manifestation of various underlying conditions, such as certain genetic disorders. Chronic atrial and intestinal dysrhythmia (CAID) is one of the rare causes of CIPO. This syndrome is an autosomal recessive cohesinopathy in which the SGOL1 (K23E) mutation disrupts cardiac and intestinal pacemaker cells, leading to sick sinus syndrome (SSS) and chronic intestinal pseudo‐obstruction (CIPO). In this case report, we present a 10‐year‐old male patient with severe gastrointestinal symptoms. After conducting all diagnostic procedures, the patient was diagnosed with Chronic Intestinal Pseudo‐Obstruction (CIPO), and further genetic testing confirmed CAID as the underlying cause of his condition. Pharmacological treatment was administered, and a clinically significant response was observed. When other causes are ruled out, and the patient also exhibits cardiac symptoms, we should consider CAID. We recommend considering genetic counseling for investigating underlying causes, such as CAID, in cases of CIPO.

## Key Clinical Message

Chronic intestinal pseudo‐obstruction (CIPO) is diagnosed through the exclusion of mechanical bowel obstruction. The management of CIPO is largely supportive, with surgical intervention not constituting first‐line treatment. Genetic etiologies may underlie CIPO, warranting genetic counseling and evaluation. The rare genetic disorder known as CAID represents one potential genetic cause of this condition.

## 1 Introduction

Chronic intestinal pseudo‐obstruction (CIPO) is a rare and serious digestive disorder that involves the disturbance of regular movement in the intestines. It mimics the symptoms of a mechanical obstruction, even though there is no physical obstruction present. This condition can occur spontaneously or as a result of other underlying medical conditions, such as mitochondrial disorders. The clinical presentation of CIPO can result from mutations in various genes, highlighting the significant genetic heterogeneity associated with this disorder [1]. Chronic atrial intestinal dysrhythmia (CAID, OMIM#616201) is one of the extremely rare causes of CIPO. This syndrome is an autosomal recessive cohesinopathy in which the SGOL1 (K23E) mutation disrupts cardiac and intestinal pacemaker cells, leading to sick sinus syndrome (SSS) and chronic intestinal pseudo‐obstruction (CIPO) [2]. In this report, we introduce a patient with CIPO who was finally diagnosed with CAID.

## 2 Case History and Examination

A 10‐year‐old boy was referred to the gastroenterology department of Mofid Children's Hospital in Tehran with complaints of abdominal pain, recurrent vomiting, and abdominal distension. The symptoms of the disease were episodic, with each episode lasting from a few days to several weeks, and the child was asymptomatic between episodes. During the episodes, the patient's symptoms improved with the use of metronidazole (10 mg/kg/dose‐TDS), rifaximin (15 mg/kg/day divided in 3 doses), and domperidone (0.2 mg/kg/dose TDS). The patient had no history of abdominal surgery. The patient didn't show any symptoms of heart disease. He had a history of hypothyroidism, which was being treated with levothyroxine, and their thyroid hormones were within the normal range. However, the patient's gastrointestinal symptoms were not related to their thyroid status, as the gastrointestinal symptoms remained unchanged during both hypothyroid and euthyroid periods. On abdominal examination, the abdomen was distended, and there were no signs of peritoneal irritation. The bowel sound was increased.

## 3 Differential Diagnosis, Investigations, and Treatment

**FIGURE 1:**
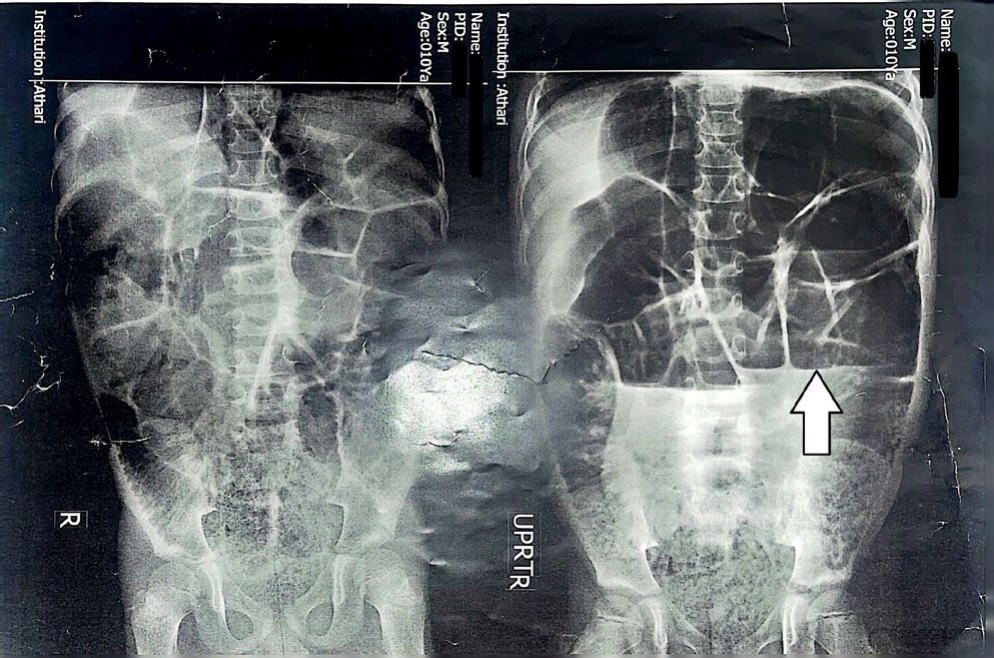
Abdominal X‐ray shows dilated loops of colon with an air–fluid level (white arrow).

**FIGURE 2:**
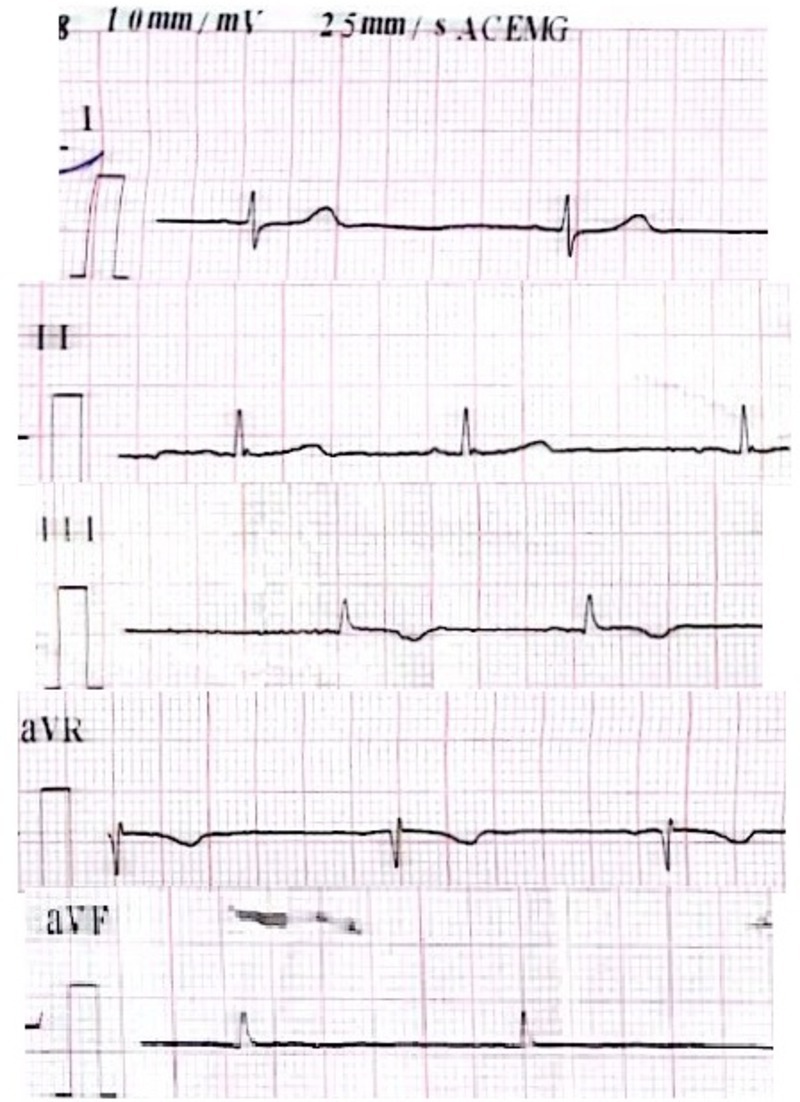
ECG shows bradycardia and arrhythmia.

Comprehensive laboratory tests were performed, and all parameters were within normal range, except for thyroid function tests, which indicated hypothyroidism. Upper gastrointestinal endoscopy revealed erosive gastritis, and the pathology report indicated mild inactive chronic gastritis and mild active duodenitis with increased eosinophils. Given the endoscopy and duodenal biopsy results, celiac disease was ruled out. In abdominal X‐ray imaging, evidence of dilatation and distension of the small intestine, along with the presence of an air‐fluid level, was observed (Figure 1). The CT scan showed dilated loops of the small intestine suggestive of intestinal obstruction without any mechanical cause. Finally, the patient was diagnosed with chronic intestinal pseudo‐obstruction (CIPO). Further investigations were requested to find the underlying cause of CIPO. Echocardiography revealed isolated supravalvular pulmonary stenosis with a moderate gradient (40 mmHg). The 24‐h Holter monitoring results showed that the patient had some PVC and PAC and a few pauses up to 2,346 ms. The average heart rate during 24 h was 66 bpm, and 6 episodes of bradycardia were recorded (Figure 2). Genetic counseling was performed, and it was found that the parents had a fourth‐degree consanguineous relationship. Whole‐exome sequencing genetic testing showed multiple variants. A homozygous missense variant was identified in the SGO1 gene (c.67A.G p.Lys23Glu). This variant is supportive for diagnosis of CAID. Based on the genetic test results and echocardiography findings, the patient was diagnosed with chronic atrial and intestinal dysrhythmia.

## 4 Conclusion and Results (Outcome and Follow‐Up)

The patient was treated with cyclic rifaximin and propranolol, which resulted in a good clinical response, and the patient's symptom attacks decreased dramatically. At the time of writing the article, the patient had not experienced any episodes of gastrointestinal issues, and the gastrointestinal symptoms had improved. The patient's growth and development had normalized, their quality of life had improved, and they were able to continue their studies without any problems.

## 5 Discussion

Chronic intestinal pseudo‐obstruction is a manifestation of intestinal dysmotility [1, 3]. Due to its rarity, limited understanding of the disease, and the absence of clearly defined diagnostic criteria, the diagnosis of CIPO is frequently delayed [4]. The diagnosis of CIPO relies primarily on clinical evaluation. The diagnostic process for patients suspected of CIPO aims to achieve the following objectives: (1) Exclude mechanical causes of bowel obstruction using imaging; (2) Identify any underlying diseases through comprehensive laboratory tests; (3) Evaluate the possibility of drug‐induced CIPO‐like symptoms, such as those caused by opioids, tricyclic antidepressants, anti‐cholinergic agents, anti‐Parkinsonian agents, or phenothiazines; and (4) Understand the pathophysiological characteristics that may guide treatment decisions or provide prognostic information, particularly by performing gastrointestinal (GI) manometry in cases without bowel dilation [5]. Abdominal pain and distension are commonly reported symptoms, along with nausea, vomiting, and constipation. Diarrhea, although less common, may occur. Typically, the symptoms of CIPO are intermittent, with periods of minimal symptoms following acute episodes. However, symptoms tend to become more persistent over time [6]. The current treatment approach for CIPO focuses on several key aspects, including restoring fluid and electrolyte balance, improving nutritional status, facilitating coordinated intestinal motility, and managing complications such as sepsis, small intestine bacterial overgrowth (SIBO), and other related symptoms. However, treating CIPO poses significant challenges and often yields unsatisfactory outcomes for both patients and physicians [6, 7].

In recent years, multiple genes have been discovered in various subsets of CIPO patients [1]. A single shared homozygous founder mutation in SGOL1, a component of the cohesin complex, is responsible for causing Chronic atrial and intestinal dysrhythmia (CAID) syndrome. In our patient, a mutation was observed in the SGOL1 gene. Many genetic disorders tend to manifest in families where the parents have a consanguineous relationship. In this case, the parents had a fourth‐degree consanguineous relationship, and this relationship was significant in the onset of this disorder in their child. CAID is a rare genetic disorder characterized by progressive dysfunction in cardiac and intestinal contractions. While cardiac arrhythmia can be managed with a pacemaker, there is currently no effective treatment or medical procedure for the debilitating intestinal dysfunction or chronic intestinal pseudo‐obstruction (CIPO) associated with CAID. Therefore, the ultimate goal is to comprehend the underlying pathomechanism to develop a treatment approach. Additionally, this presents an opportunity to investigate CIPO and intestinal pseudo‐obstruction more broadly, capitalizing on the fact that a single genetic mutation is responsible for the manifestation of the disease [8].

One of the very rare causes of CIPO is CAID. When other causes are ruled out, and the patient also exhibits cardiac symptoms, we should consider CAID. We recommend considering genetic counseling for investigating underlying causes, such as CAID, in cases of CIPO.50.

## Author Contributions

**Amirhossein Hosseini:** conceptualization, investigation, methodology, supervision, writing – review and editing. **Mina Alibeik:** data curation, resources, visualization, writing – original draft, writing – review and editing. **Soheil Omid:** formal analysis, software, writing – original draft. **Koroush Vahidshahi:** supervision, visualization. **Soheila Vaghefi:** supervision. **Aliakbar Sayyari:** supervision, visualization. **Farid Imanzadeh:** resources, validation.

## Funding

The authors have nothing to report.

## Consent

Although this report does not contain any personal information that could lead to the identification of the patient, written informed consent has been obtained from the patient's parents.

## Data Availability

The data that support the findings of this case report are derived from the patient's hospital records at Mofid Hospital in Tehran, along with other relevant documentation. These records are archived at the hospital and are available only to the treatment team of the hospital upon obtaining the necessary permissions.
